# Elamipretide(SS-31) Attenuates Idiopathic Pulmonary Fibrosis by Inhibiting the Nrf2-Dependent NLRP3 Inflammasome in Macrophages

**DOI:** 10.3390/antiox12122022

**Published:** 2023-11-21

**Authors:** Yunjuan Nie, Jiao Li, Xiaorun Zhai, Zhixu Wang, Junpeng Wang, Yaxian Wu, Peng Zhao, Gen Yan

**Affiliations:** 1 Department of Basic Medicine, Wuxi School of Medicine, Jiangnan University, Wuxi 214122, China; nieyunjuan@jiangnan.edu.cn (Y.N.); 6212805004@stu.jiangnan.edu.cn (J.L.); 6222809142@stu.jiangnan.edu.cn (Z.W.); 1282200125@stu.jiangnan.edu.cn (J.W.); yaxianwu@jiangnan.edu.cn (Y.W.); 2 Department of Radiology, The Second Affiliated Hospital of Xiamen Medical College, Xiamen 361000, China

**Keywords:** SS-31, IPF, macrophage, NLRP3 inflammasome, Nrf2

## Abstract

Idiopathic pulmonary fibrosis (IPF) is a progressive fatal lung disease with a limited therapeutic strategy. Mitochondrial oxidative stress in macrophages is directly linked to IPF. Elamipretide(SS-31) is a mitochondrion-targeted peptide that has been shown to be safe and beneficial for multiple diseases. However, whether SS-31 alleviates IPF is unclear. In the present study, we used a bleomycin (BLM)-induced mouse model followed by SS-31 injection every other day to investigate its role in IPF and explore the possible mechanism. Our results showed that SS-31 treatment significantly suppressed BLM-induced pulmonary fibrosis and inflammation, with improved histological change, and decreased extracellular matrix deposition and inflammatory cytokines release. Impressively, the expression percentage of IL-1β and IL-18 was downregulated to lower than half with SS-31 treatment. Mechanistically, SS-31 inhibited IL-33- or lipopolysaccharide(LPS)/IL-4-induced production of IL-1β and IL-18 in macrophages by suppressing NOD-like receptor thermal protein domain associated protein 3(NLRP3) inflammasome activation. Nuclear factor erythroid 2-related factor 2(Nrf2) was dramatically upregulated along with improved mitochondrial function after SS-31 treatment in activated macrophages and BLM-induced mice. Conversely, there was no significant change after SS-31 treatment in Nrf2−/− mice and macrophages. These findings indicated that SS-31 protected against pulmonary fibrosis and inflammation by inhibiting the Nrf2-mediated NLRP3 inflammasome in macrophages. Our data provide initial evidence for the therapeutic efficacy of SS-31 in IPF.

## 1. Introduction

IPF is a fatal lung disease characterized by a wound-healing cascade that does not resolve, leading to chronic inflammation, exaggerated scarring, loss of lung function, and ultimately death [1]. IPF has an increasing prevalence of over 60 per 100,000 persons, while current therapies for this devastating disorder have been largely unsuccessful, even with extensive studies [2]. Hence, IPF has a poor prognosis with a mean survival time within 2 to 3 years after diagnosis [3]. There is an urgent need to develop novel therapeutic agents to prevent and manage IPF.

Macrophages are the most abundant immune cells, which play a central role during IPF development [4]. With highly plastic characteristics, macrophages adopt different activation states according to a stimulus [5]. Macrophage polarization refers to the mechanism by which macrophages differentiate into a series of different phenotypes under different environmental stimuli, exhibit a wide range of functions, and exert immune effects in local environments and signals. Generally, macrophages polarize into the classically activated (M1) phenotype in response to stimulation with the Th1 mediators interferon-γ (IFN-γ) and lipopolysaccharide (LPS) [6] but into the alternatively activated (M2) phenotype with induction of the Th2 cytokines IL-4 and IL-13 [7]. It is widely believed that macrophage activation occurs throughout IPF development from the early inflammatory phase to the subsequent fibrotic phase [5]. M1 macrophages promote inflammation by secreting proinflammatory cytokines, such as TNFα, IL-6, and IL-1β [8], and M2 macrophages can secrete profibrotic factors, such as transforming growth factor β (TGF-β) and platelet-derived growth factor (PDGF), to induce continuous fibroblast proliferation and differentiation and finally develop fibrosis [9]. Recently, several studies have revealed that the immune environment during IPF is usually complicated by both Th1 and Th2 cytokines, which simultaneously act on macrophages to promote their activation and final fibrosis [10,11]. Additionally, IL-33 is another important cytokine that is released from injured epithelial cells and signals via the ST2 receptor during the wound-healing process. To date, the IL-33-ST2 signaling axis has been found to play a central role in driving macrophage activation, leading to the release of various cytokines, including TNF-α, IL-1β, TGF-β, and IL-13, and finally resulting in fibrogenesis in the lung [12,13,14]. Therefore, macrophages in IPF show a mixed phenotype and simple therapeutic agents designed to repress M1 or M2 macrophages may be insufficient. Intervention for macrophage activation in response to IL-33 or the complex of Th1 and Th2 cytokines might be a more effective way to protect against IPF. 

Repetitive lung injury caused by various reasons can lead to macrophage activation, fibroblast proliferation, and tissue remodeling leading to pulmonary fibrosis. In the development process of IPF, mitochondrial dysfunction and oxidative stress in alveolar macrophages are important conditions for fiber production, among which mitochondrial ROS play a crucial role in the metabolic changes in IPF [15]. Mitochondria are important signaling organelles that serve as central players in macrophage activation under various stimuli [16]. There is growing evidence showing that abnormal macrophage activation can be induced by mitochondrial dysfunction and subsequent accumulation of mitochondrial ROS (mtROS), damaged mitochondrial DNA (mtDNA), and mitochondria-associated proteins [17]. Therefore, the loss of mitochondrial homeostasis plays a central role in the pathophysiology of inflammatory diseases, and mitochondrion-targeted interventions have received considerable attention for various diseases related to inflammation [18,19]. In IPF, accumulated dysmorphic and dysfunctional mitochondria have been noted in the lungs, resulting in a maladaptation to cellular stress and creating a vulnerable environment to injury that contributes to the development of pulmonary fibrosis. SS-31 is a novel and promising mitochondrial-targeting synthetic compound that can eliminate mtROS and damaged mtDNA, thus restoring mitochondrial membrane potential and inhibiting oxidative stress [20,21]. It has been reported that SS-31 has numerous advantages, making it attractive for future clinical applications. For example, SS-31 is a small polypeptide that is water-soluble and not easily degraded by peptidase; SS-31 can not only undergo cellular uptake but also be transported across cells, thus being absorbed rapidly and exerting functions after administration [22]. Accordingly, SS-31 was found to protect against a series of diseases, such as atherosclerosis, heart failure, and renal fibrosis, in experimental studies by improving mitochondrial function by reducing mitochondrial ROS emission and blocking mitochondrial membrane potential loss [23,24,25]. Recently, SS-31 was demonstrated to effectively suppress macrophage activation under certain conditions and be beneficial for various inflammatory diseases [26,27]. For example, SS-31 can suppress M1 macrophage polarization through ROS scavenging and STAT1/STAT3 signaling to attenuate ischemia-reperfusion injury [27]; in a rat model of bilateral renal ischemia, SS-31 treatment significantly reduced macrophage infiltration, ameliorated the expression levels of proinflammatory cytokines, and restored glomerular capillaries and podocyte structure [26]. This effective role of SS-31 brought the dawn of the treatment of IPF, whose pathogenesis is closely related to macrophage activation and mitochondrial dysfunction. However, the roles of SS-31 in macrophage alterations in complex immune conditions during IPF progression have never been explored.

In the current study, we confirmed for the first time the antifibrotic activity of SS-31 in IPF through antagonizing macrophage activation and revealed a novel molecular mechanism accounting for the therapeutic effect of SS-31 on pulmonary fibrosis. This study may provide a new treatment strategy for IPF by making mitochondria a potential target.

## 2. Materials and Methods

### 2.1. Mice

C57BL/6 mice were purchased from SLAC Laboratory Animal Corporation (Shanghai, China). Nrf2-deficient (Nrf2−/−) mice were kindly provided by Professor Qing-feng Pang (Jiangnan University, Wuxi, China) and were backcrossed for >11 generations on the C57BL/6 background. Five mice in each cage were housed in specific pathogen-free conditions in a climate-controlled room (25 °C, 55% humidity, and a 12-hour light/darkness cycle). The procedures involving mice were approved by the Instructional Animal Care and Use Committee of Jiangnan University (JN. No20211130m1720615, approval date 22 December 2021).

### 2.2. Reagents

SS-31 (C32H49N9O5, CAS: 736992-21-5, purity = 98.53%,) was purchased from Target Molecule Corp, Ltd. (Shanghai, China). Bleomycin was purchased from BioTang (Lexington, MA, USA). Primary antibodies against α-smooth muscle actin (α-SMA, #19245), NLRP3 (#20836), caspase-1 (#20836), apoptosis-associated speck-like protein containing CARD (ASC, #20836), and Nrf2 (#12721) were purchased from Cell Signaling Technology (Danvers, MA, USA), and β-actin (#21338) was purchased from Signalway Antibody (College Park City, MD, USA).

### 2.3. BLM-Induced Pulmonary Fibrosis

Male mice (20–25 gm) aged 6–8 weeks in good condition were randomly divided into 4 groups: saline + saline group, BLM + saline group, BLM + SS-31 (5 mg/kg) group, and SS-31 alone (5 mg/kg) group. Each group included 6–8 mice. SS-31 was diluted with saline. After anesthesia with pentobarbital sodium, mice were intratracheally (i.t.) injected with BLM (BioTang; 1.4 U/kg) or an equal volume of saline on day 0. The indicated dosage of SS-31 was intraperitoneally (i.p.) administered every other day beginning on day 1, and the same volume of saline was i.p. administered at the same time point in the group without SS-31 treatment. The saline + SS-31 group was used as a sham group. All mice were euthanized on days 3, 7, and 21 after BLM exposure; bronchoalveolar lavage and lung tissues were collected immediately for subsequent studies.

### 2.4. Bronchoalveolar Lavage Fluid (BALF) Collection and Analysis

BALF was collected from the indicated groups on days 3 and 7 by cannulating the trachea and lavaging the lung with 1 mL of sterile PBS twice. BALF was centrifuged at 4 °C and 500× *g* for 5 min. Total cells in the BALF pellet were washed, resuspended in PBS, and counted. Cell-free BALF supernatant was analyzed for protein concentration using the BCA protein assay kit (Beyotime Biotechnology, Shanghai, China).

### 2.5. Lung Histological Assay

On days 3, 7, and 21 after BLM administration, mice were sacrificed, and lung tissues (left lobe) were fixed in 4% paraformaldehyde before being embedded in paraffin and then cut into 4 μm thick sections. The slides were stained with hematoxylin and eosin (H&E) to evaluate inflammatory and fibrotic injury of inflammatory cells. The degree of lung injury was assessed and graded from 0 (normal) to 4 (severe), as previously described, in a blinded manner [28]. Masson trichrome staining was conducted following the instructions of Nanjing Jiancheng Bioengineering Institute, Nanjing, China, to evaluate collagen expression in lung tissues on day 21. The criteria of Ashcroft’s assay were applied to slides from day 21 for scoring pulmonary fibrosis.

### 2.6. Hydroxyproline Assay

The right lungs of mice were isolated and hydrolyzed, and hydroxyproline levels were measured using a hydroxyproline assay kit (A030-2-1, Nanjing Jiancheng Bioengineering Institute, Nanjing, China). Each sample was tested in triplicate. Data were expressed as micrograms of hydroxyproline per gram lung.

### 2.7. Primary Macrophage Culture and Treatment

Bone marrow-derived macrophages (BMDMs) and lung macrophages were obtained from male mice as we previously published [12]. Briefly, BMDMs were collected from the femurs and tibia of mice and cultured in complete Dulbecco’s modified eagle medium (DMEM) in the presence of 10% FBS and macrophage colony-stimulating factor (M-CSF) (10 ng/mL, Novoprotein ShuZhou, China), which was replaced with fresh DMEM containing M-CSF on the fourth day. Then, differentiated BMDMs were collected for experiments on the seventh day; lung macrophages were isolated from the whole lungs of mice with collagenase digestion (1.0 mg/mL collagenase, 25 U/mL DNase). Cells were collected and cultured in DMEM plus 10% FBS at 37 °C in a humidified atmosphere incubator with 5% CO_2_ for 1 h. Then, the nonadherent cells were washed away, and the adherent cells were used for experiments. For the in vitro experiments, cells were divided into 3 groups: saline group as control, IL-33 or LPS/IL-4 stimulation group, IL-33 or LPS/IL-4 stimulation + SS-31 (10 μM SS-31 was treated 15 min after the stimulants were given) group, and SS-31 alone (10 μM) group. The doses for cell stimulation were 10 ng/mL for IL-33 and IL-4 and 100 ng/mL for LPS. Samples were collected for subsequent studies after treatment.

### 2.8. Isolation of Total RNA and Quantitative PCR

Total RNA from lung tissues and macrophages was isolated using TRIzol reagent (Vazyme Biotech Co., Ltd, Nanjing, China). cDNA was prepared with a ReverTra Ace qPCR RT Kit (Toyobo, Osaka, Japan). The mRNA expression of target genes was determined with real-time PCR with SYBR Green Master Mix (Toyobo, Osaka, Japan) on a LightCycler^®^ 480 PCR detection system (AXYPCR96LC480 WNF, Roche, Foster City, CA, USA). The gene accession number of Nrf2 is NM_010902.5. The primer sequences are listed below:
Target genePrimerPrimer sequences (5′ -3′):α-SMAForwardGACGCTGAAGTATCCGATAGAACACG;Reverse
CACCATCTCCAGAGTCCAGCACAAT.*Collagen I*ForwardTGCCGTGACCTCAAGATGTG;Reverse
CACAAGCGTGCTGTAGGTGA.*Fibronectin*ForwardTCTGGGAAATGGAAAAGGGGAATGG;Reverse.CACTGAAGCAGGTTTCCTCGGTTGT*TNF-α*ForwardTTCTCAT TCCTGCTTGTGG;Reverse
ACTTGGTGGTTTG CTACG.*IL-1β*ForwardCCAGCTTCAAATCTCACAGCAG;Reverse
CTTCTTTGGGTATTGCTTGGGATC.*IL-6*ForwardCCACCAAGAACGATAGTCAA;Reverse
TTTCCACGATTTCCCAGA.*Nrf2*ForwardCCCGAAGCACGCTGAAGGCA;Reverse
CCAGGCGGTGGGTCTCCGTA.*mtDNA*ForwardGGTTCTTACTTCAGGGCCATCA;Reverse
GATTAGACCCGTTACCATCGAGAT.*Nrf1*ForwardCCATCTATCCGAAAGAGACAGC;Reverse
GGGTGAGATGCAGAGTACAATC.*IL-18*ForwardACTTTGGCCGACTTCACTGT;Reverse
GGGTTCACTGGCACTTTGAT.*NLRP3*ForwardGCTGTGTGAGGCACTCCAG;Reverse
GGAGATGTCGAAGCAGCATT.*ASC*ForwardGGAGTCGTATGGCTTGGAGC;Reverse
CGTCCACTTCTGTGACCCTG.*Caspase-1*ForwardCCCACTGCTGATAGGGTGAC;Reverse
GCATAGGTACATAAGAATGAACTGGA.*GAPDH*ForwardTGCGACTTCAACAGCAACTC;Reverse
CTTGCTCAGTGTCCTTGCTG.

### 2.9. Western Blotting

Total protein from lung tissues and treated cells were lysed and extracted with 1× RIPA lysis buffer. The protein concentration was measured using a BCA Protein Assay Reagent kit (Beyotime Biotechnology, Shanghai, China). Equal amounts of proteins (20 μg) were separated on 12% SDS–PAGE gels and transferred to nitrocellulose membranes (Millipore, Billerica, MA, USA). After being blocked with 5% BSA for 1 h at room temperature, the proteins were incubated with target primary antibodies (1:1000) overnight at 4 °C and HRP-conjugated secondary antibodies for 2 h at room temperature, and then visualized with an ECL kit (Millipore, Billerica, MA, USA) using a ChemiDoc MP Imaging System (Bio-Rad Laboratories, Hercules, CA, USA). Quantification of the bands was performed with ImageJ software (ImageJ-Java 1.8.0_172(164-bit), Software Inquiry; Quebec, QC, Canada).

### 2.10. Immunofluorescence Assay

Macrophages (2 × 10^4^/well) were plated in 48-well plates containing coverslips and treated with the indicated treatments. Then, the cells were fixed in 4% paraformaldehyde for 10 min and permeabilized with 0.1% Triton X-100(Sigma Aldrich, Shanghai, China). After being blocked with 5% BSA for 30 min, the cells were incubated with the target primary antibody overnight at 4 °C. After removing the primary antibodies and washing three times with PBS, the cells were further incubated with Alexa 488-labeled secondary antibody (CST, Danvers, MA, USA) for 2 h and DAPI(4′,6-diamidino-2-phenylindole) (2.5 μg/mL) for 5 min. Then, the coverslips were transferred onto glass substrates and photographed under a laser-scanning confocal fluorescence microscope (TCS SP8, Leica Microsystem, Wetzlar, Germany).

### 2.11. Determination of Cellular Reactive Oxygen Species (ROS)

To determine cellular ROS, macrophage cells were pretreated with IL-33/LPS + IL-4 treatment and then incubated with 10 μM oxidation-sensitive fluorescent probe DCFH-DA (Beyotime, Shanghai, China) in a dark room for 20 min at 37 °C. Then, the cells were photographed under a laser-scanning confocal fluorescence microscope (TCS SP8, Leica Microsystem, Wetzlar, Germany). The average level of ROS in cells was quantitatively analyzed with fluorescence using ImageJ. ImageJ-Java 1.8.0_172(164-bit) National Institutes of Health, Bethesda, MD, USA.

### 2.12. Statistical Analysis

Data are expressed as the mean ± SEM. The in vitro data were obtained from at least three independent experiments, and the in vivo data were obtained from experiments with 6–8 mice in each group. Statistical differences between single comparisons were performed using Student’s *t*-test, and multiple groups were compared using nonparametric ANOVA and a Kruskal–Wallis test using GraphPad Prism 9. Statistical significance was defined as * *p* < 0.05, ** *p* < 0.01, and *** *p* < 0.001. 

## 3. Results

### 3.1. SS-31 Attenuated BLM-Induced Pulmonary Fibrosis

**Figure 1:**
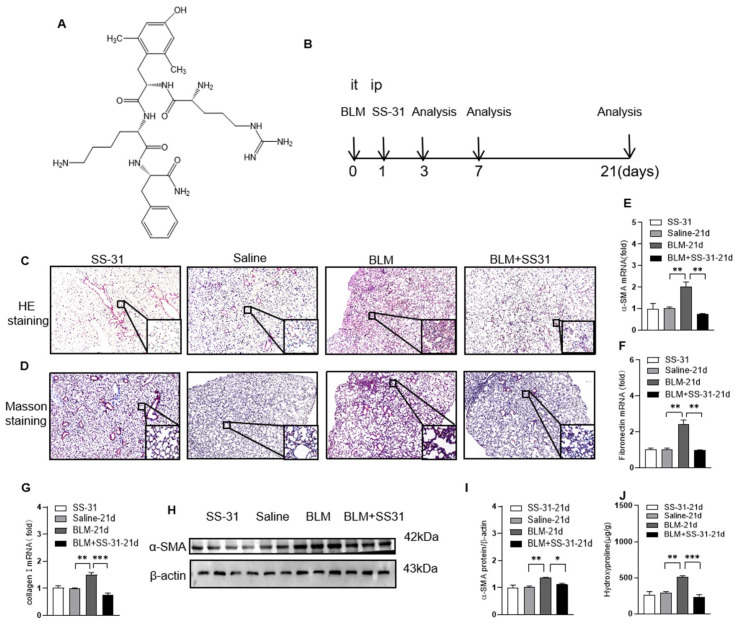
Administration of SS-31 ameliorated BLM-induced pulmonary fibrosis in mice. (**A**) Chemical structure. (**B**) Schematic diagram for the use of BLM and SS-31 described in the Materials and Methods. Simply, C57BL/6 mice were i.t. challenged with saline or BLM (1.4 U/kg) followed by i.p. injection of SS-31 (5 mg/kg) or dilution every other day. After 21 days, lung tissue sections were performed with (**C**) H&E staining and (**D**) Masson trichrome staining (original magnification ×400, the scale bar is 100 μm). (**E**–**G**) RNA was extracted from lung tissues, and the mRNA levels of α-SMA and fibronectin were detected using RT-qPCR. (**H**,**I**) The protein level of α-SMA, collagen was analyzed using Western blotting and quantified using ImageJ software. ImageJ-Java 1.8.0_172(164-bit) (**J**) Hydroxyproline was detected in different groups of mice. Data are shown as mean ± SEM. N = 6–8 mice for each group; multiple group non-parametric ANOVA and Kruskal–Wallis test, * *p* < 0.05, ** *p* < 0.01, *** *p* < 0.001.

The chemical structure of SS-31 is shown in Figure 1A. To evaluate the role of SS-31 in pulmonary fibrosis, BLM-induced IPF mice were treated with SS-31 (5 mg/kg diluted in saline) or control dilution by i.p. injection every other day. The state of mice in different groups was observed, and no marked change in the hair, respiration, behavior, or weight was observed in the different groups. Lung samples were collected for analysis 21 days later (Figure 1B). In the BLM group, H&E staining showed worsened pulmonary alveolar structure remodeling and accumulated fibrotic lesions, and Masson staining showed obvious destruction with increased collagen deposition (blue area), while SS-31 treatment markedly reduced the above fibrotic conditions and collagen deposition. No significant SS-31 toxicity was observed at the indicated concentration from the histologic assay (Figure 1C,D). Consistently, RT–qPCR and Western blot analysis indicated that the increased expression of the fibrogenic markers a-SMA, fibronectin, and collagen I in the lungs of BLM-challenged mice was significantly downregulated after SS-31 treatment (Figure 1E–I). We also determined the hydroxyproline content in the lung homogenates, a marker correlated with fibrosis severity, and found that the hydroxyproline level in BLM-induced mice was decreased with SS-31 treatment (Figure 1J). Taken together, these results indicate that SS-31 protects mice against BLM-induced lung fibrosis.

### 3.2. SS-31 Attenuated BLM-Induced Pulmonary Inflammation and Reduced the Expression of IL-1β and IL-18 In Vivo

**Figure 2:**
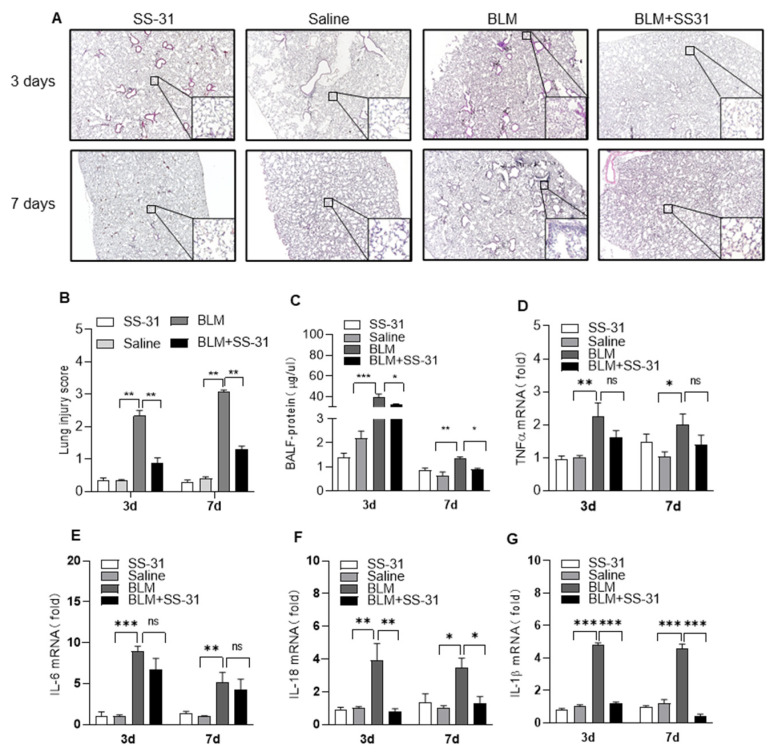
Administration of SS-31 ameliorated BLM-induced pulmonary inflammation in mice. C57BL/6 mice were i.t. challenged with saline or BLM (1.4 U/kg) for 3 and 7 days followed by i.p. injection of SS-31 (5 mg/kg) every other day. (**A**,**B**) Lung tissue sections were performed with H&E staining (the scale bar is 100 μm). (**C**) Alveolar damage and protein leak into BALF was determined by the protein concentration. (**D**–**G**) The mRNA level of TNF-α, IL-6, IL-1β, and IL-18 in lung tissues on days 3 and 7 was quantified using qRT-PCR. Data are shown as mean ± SEM. N = 6–8 mice for each group, * *p* < 0.05, ** *p* < 0.01, *** *p* < 0.001.

The inflammatory response is an important trigger for pulmonary fibrosis. We next explored whether the anti-fibrotic efficacy of SS-31 was attributed to its anti-inflammatory activity in the early stage. H&E staining showed that SS-31 significantly reduced the infiltration of inflammatory cells and promoted the integrity of organizational structures in BLM-challenged mice (Figure 2A). The degree of lung injury was further evaluated with a semiquantitative score, and SS-31 treatment significantly reduced BLM-induced lung injury scores (Figure 2B). Lung alveolar microvascular permeability can be reflected by the protein concentration in BALF. The results showed markedly lower protein concentrations in BALF after SS-31 treatment in BLM-induced mice (Figure 2C). Lung injury is usually accompanied by the release of inflammatory cytokines, and we next measured whether SS-31 could reduce the expression of inflammatory genes in BLM-treated mice. Interestingly, SS-31 treatment specifically reduced the expression of IL-1β and IL-18 in lung tissues, and some other inflammatory factors, such as tumor necrosis factor-α (TNF-α), IL-6, and iNOS, showed a slight but not significant reduction (Figure 2D–G), suggesting that SS-31 alleviated BLM-induced lung inflammation by specifically reducing IL-1β and IL-18.

### 3.3. SS-31 Inhibited NLRP3 Inflammasome Activation in Macrophages and BLM-Induced Mice

**Figure 3:**
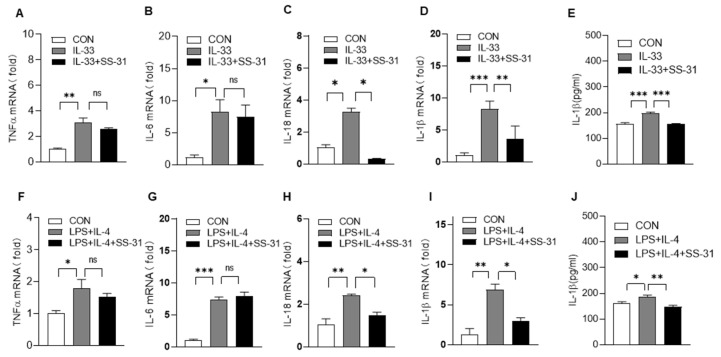
SS-31 reduced the expression of IL-1β and IL-18 in macrophages. BMDMs were induced with IL-33 (10 ng/mL), followed by treatment with saline or SS-31(10 nM); 24 h later, cell samples were collected. (**A**–**D**) The mRNA level of TNF-α, IL-6, IL-18, and IL-1β was detected using qRT-PCR. (**E**) The protein level of IL-1β was detected using an ELISA assay; BMDMs were induced with LPS (100 ng/mL) and IL-4 (10 ng/mL), followed by treatment with saline or SS-31(10 nM) for 24 h, and then cell samples were collected. (**F**–**I**) The mRNA level of TNF-α, IL-6, IL-18, and IL-1β was detected using qRT-PCR. (**J**) The protein level of IL-1β was detected using an ELISA assay. Each experiment was independently repeated in triplicate with duplicated wells. Data represent means ± SEM, * *p* < 0.05, ** *p* < 0.01, *** *p* < 0.001, ns >= 0.05.

**Figure 4:**
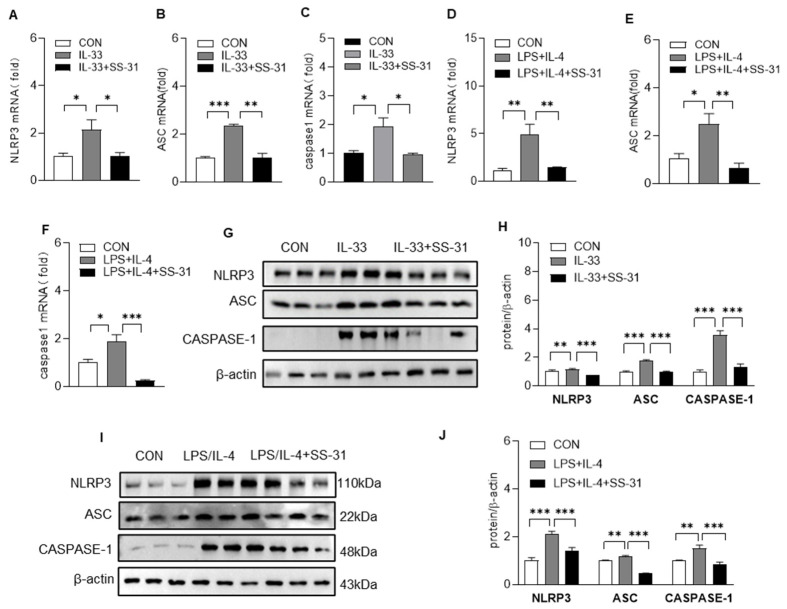
SS-31 alleviated NLRP3 inflammasome activation in macrophages. BMDMs were induced with IL-33 (10 ng/mL), LPS (100 ng/mL) plus IL-4 (10 ng/mL), followed with a treatment of saline or SS-31(10 nM) for 24 h, and then cell samples were collected. (**A**–**F**) The mRNA level of NLRP3, ASC, and caspase-1 was detected using qRT-PCR. (**G**,**H**) BMDMs were induced with IL-33 (10 ng/mL), and the protein level of NLRP3, ASC, and caspase-1 was analyzed using Western blotting quantified using ImageJ software. (**I**,**J**) BMDMs were induced with LPS (100 ng/mL) plus IL-4 (10 ng/mL), and the protein level of NLRP3, ASC, and caspase-1 was analyzed using Western blotting and quantified using ImageJ software. Data represent means ± SEM of three independent experiments, * *p* < 0.05, ** *p* < 0.01, *** *p* < 0.001.

**Figure 5:**
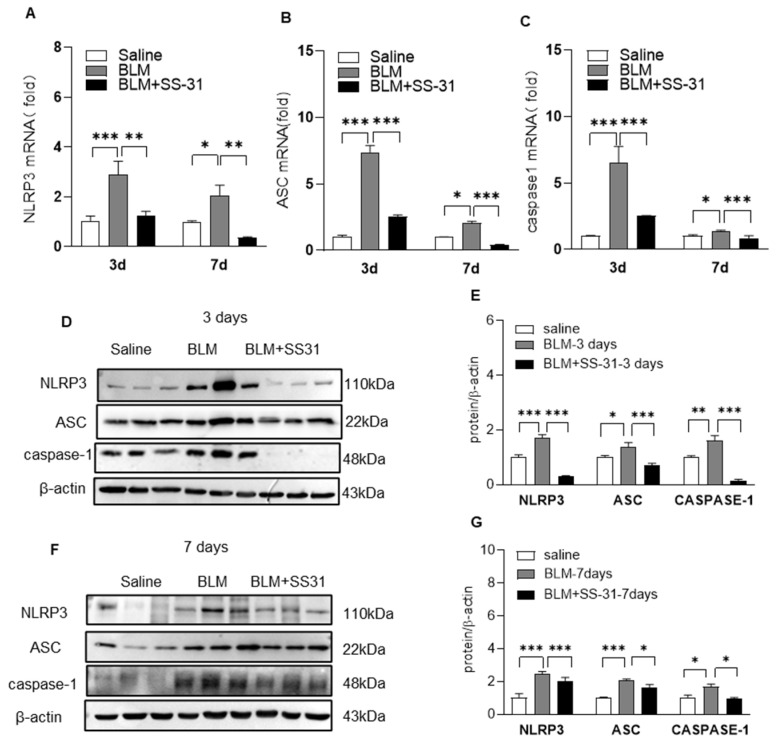
SS-31 alleviated NLRP3 inflammasome activation in mice. C57BL/6 mice were i.t. challenged with saline or BLM (1.4 U/kg) for 3 and 7 days followed by i.p. injection of SS-31 (5 mg/kg) every other day. (**A**–**C**) The mRNA level of NLRP3, ASC, and caspase-1 was detected using qRT-PCR. (**D**–**G**) The protein level of NLRP3, ASC, and caspase-1 was analyzed using Western blotting and quantified using ImageJ software. Data represent means ± SEM, N = 6–8 mice for each group, * *p* < 0.05, ** *p* < 0.01, *** *p* < 0.001

Macrophages are the predominant source of proinflammatory and profibrotic mediators during the IPF process; thus, we examined the effect of SS-31 on macrophage activation. As shown in Figure 3A–J, SS-31 treatment also reduced the expression of IL-1β and IL-18 with no significant effect on inflammatory factors such as TNF-α and IL-6 in macrophages induced by IL-33 or LPS/IL-4. Previous studies have demonstrated that the NLRP3 inflammasome, which contains caspase-1, ASC, and NLRP3, is a central mediator of the secretion of IL-1β and IL-18. Here, we continued to examine the effect of SS-31 on the NLRP3 inflammasome complex using RT–qPCR and Western blotting. The results showed that both IL-33 and LPS/IL-4 stimulation could enhance the expression of NLRP3, apoptosis-associated speck-like protein containing a CARD(ASC), and cysteinyl aspartate specific proteinase-1(caspase-1) in macrophages, and SS-31 treatment inhibited the enhancement in these NLRP3 inflammasome components (Figure 4A–J). Reduced expression of the NLRP3 inflammasome was observed in the lungs of BLM-induced mice after SS-31 treatment (Figure 5A–G). Collectively, these data suggested that SS-31 reduced the release of the cytokines IL-1β and IL-18 in macrophages as well as in the lungs of IPF mice by inhibiting NLRP3 inflammasome activation.

### 3.4. SS-31 Restored NRF2 Expression and Mitochondrial Function in Macrophages

**Figure 6:**
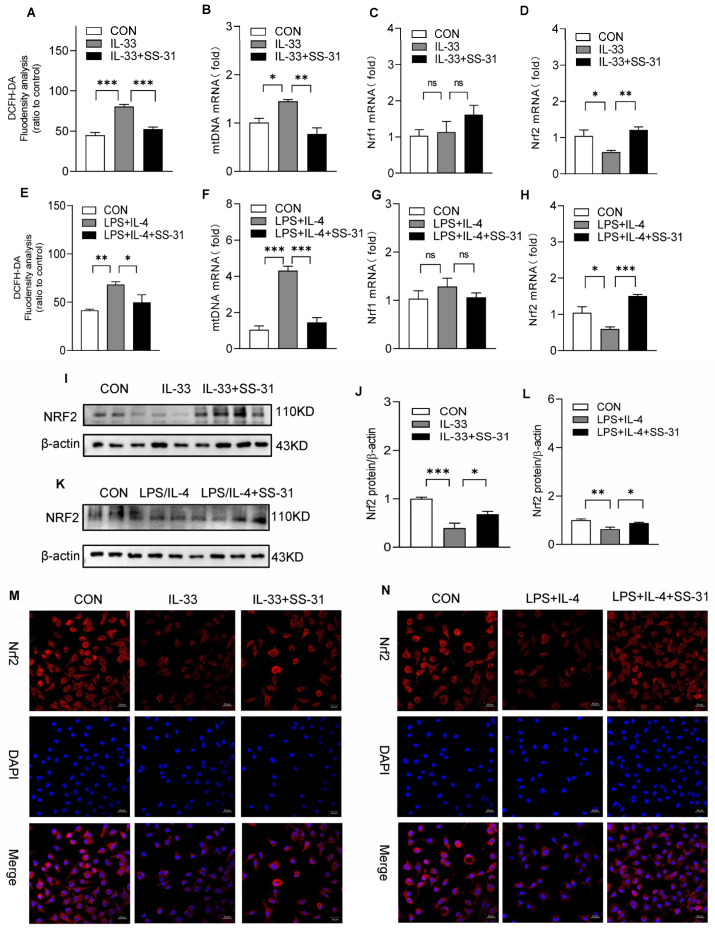
SS-31 restored mitochondrial function and Nrf2 expression. BMDMs were induced with IL-33 (10 ng/mL) and LPS (100 ng/mL) plus IL-4 (10 ng/mL), followed by treatment with saline or SS-31 (10 nM) for 24 h, and then cell samples were collected. (**A**,**E**) The level of ROS was analyzed. (**B**–**D**,**F**–**H**) The expression of mtDNA, Nrf1, and Nrf2 was detected using qRT-PCR. (**I**–**L**) The protein level of Nrf2 was analyzed using Western blotting. (**M**,**N**) The expression and translocation of Nrf2 (Red) and nuclei (blue) were detected using immunofluorescence assay. Data represent means ± SEM of three independent experiments, * *p* < 0.05, ** *p* < 0.01, *** *p* < 0.001, ns >= 0.05.

Previous studies demonstrated that mitochondrial dysfunction, the release of mtROS, and the accumulation of mtDNA in the cytosol are key upstream events implicated in NLRP3 activation. We further explored the effect of SS-31 on the mitochondria of macrophages under stimulation. The results showed that SS-31 treatment significantly inhibited the accumulation of mtDNA and the production of ROS in macrophages in response to IL-33 and LPS/IL-4 stimulation (Figure 6A,B,E,F). Considerable evidence supports that Nrf1 and Nrf2 are closely related to oxidative damage to mitochondria. Our results showed that both IL-33 and LPS/IL-4 challenge could reduce the mRNA levels of Nrf1 and Nrf2, while only the expression of Nrf2 could be restored with SS-31 treatment (Figure 6C,D,G,H). Further detection using Western blotting and immunofluorescence assays showed enhanced protein expression and nuclear translocation of Nrf2 after SS-31 treatment (Figure 6I–N). Collectively, these data indicated that Nrf2 might be involved in mitochondrial dysfunction and NLRP3 inflammasome activation in macrophages.

### 3.5. Nrf2 Knockout Reversed the Effect of SS-31 on NLRP Inflammasome Activation in Macrophages

**Figure 7:**
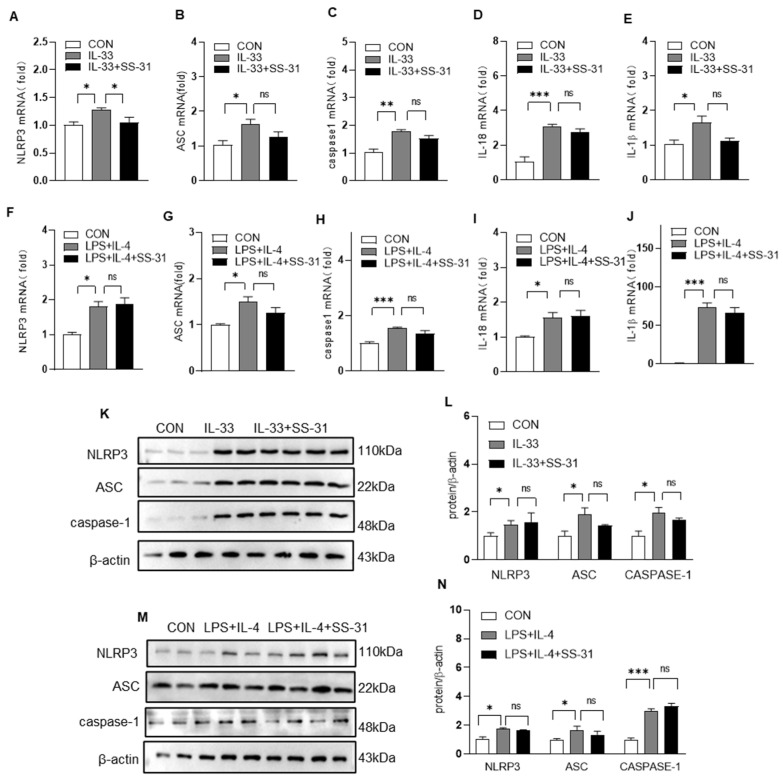
SS-31 treatment did not affect NLRP3 inflammasome in Nrf2−/− macrophages. Nrf2−/− BMDMs were induced with IL-33 (10 ng/mL) and LPS (100 ng/mL) plus IL-4 (10 ng/mL), followed by treatment with saline or SS-31 (10 nM) for 24 h, and then cell samples were collected. (**A**–**J**) The mRNA level of NLRP3, ASC, caspase-1, IL-18, and IL-1β was detected using qRT-PCR. (**K**–**N**) The protein level of NLRP3, ASC, and caspase-1 was analyzed using Western blotting and quantified using ImageJ software. Data represent means ± SEM of three independent experiments, * *p* < 0.05, ** *p* < 0.01, *** *p* < 0.001, ns >= 0.05.

To further determine whether the function of SS-31 in NLRP3 inflammasome activation and cytokine production was dependent on Nrf2 in macrophages, Nrf2−/− macrophages were stimulated with IL-33 or LPS/IL-4, followed by treatment with SS-31 or PBS as a control. The RT–qPCR and Western blotting results showed that in Nrf2-deleted macrophages, SS-31 treatment did not change the expression of NLRP3 inflammasome components, including NLRP3, caspase-1 and ASC, which were increased by IL-33 or LPS/IL-4 stimulation (Figure 7A,C,F–H,K–N). Correspondingly, the production of IL-1β and IL-18 was also not affected after SS-31 treatment in Nrf2−/− macrophages (Figure 7D,E,I,J), suggesting the key role of Nrf2 signaling in the suppression of macrophage activation by SS-31.

### 3.6. Nrf2 Knockout Reversed the Effect of SS-31 on NLRP3 Inflammasome Activation and Fibrosis in BLM-Induced Mice

**Figure 8:**
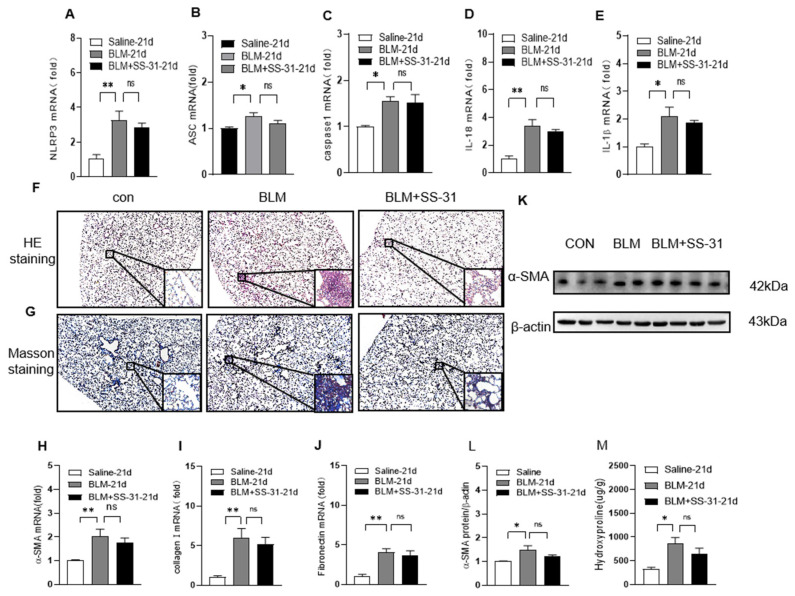
SS-31 treatment did not affect NLRP3 inflammasome and fibrosis in BLM-induced Nrf2−/− mice. Nrf2−/− mice were i.t. challenged with saline or BLM (1.4 U/kg) for 21 days followed by i.p. injection of SS-31 (5 mg/kg) every other day. (**A**–**E**) The mRNA level of NLRP3, ASC, caspase-1, IL-1β, and IL-18 was detected using qRT-PCR. (**F**,**G**) Lung tissue sections were performed using H&E staining and Masson trichrome staining (original magnification ×400, the scale bar is 100 μm). (**H**,**I**,**J**) RNA was extracted from lung tissues, and the mRNA levels of α-SMA and fibronectin were detected using RT-qPCR. (**K**,**L**) The protein level of α-SMA was analyzed using Western blotting and quantified using ImageJ software. (**M**) Hydroxyproline was detected in different groups of mice. Data represent means ± SEM, N = 6–8 mice for each group, * *p* < 0.05, ** *p* < 0.01, ns >= 0.05.

To confirm whether the role of SS-31 in NLRP3 inflammasome activation and pulmonary fibrosis was Nrf2 dependent in vivo, we detected the effect of SS-31 on BLM-induced Nrf2−/− mice. As expected, SS-31 did not change the mRNA or protein levels of NLRP3, caspase-1, or ASC (Figure 8A–C), subsequently resulting in unchanged levels of IL-1β and IL-18 in lung tissues (Figure 8D,E). Furthermore, the pathology of pulmonary fibrosis observed with H&E staining was markedly enhanced in BLM-induced Nrf2−/− mice, while SS-31 exerted no significant function in improving the pathological change (Figure 8F). Similarly, Masson trichrome staining showed that SS-31 did not reduce pulmonary collagen deposition in Nrf2−/− mice (Figure 8G). In addition, the BLM challenge in Nrf2−/− mice substantially increased the expression levels of α-SMA, fibronectin, and collagen deposition in lung sections, which could not be downregulated with SS-31 treatment (Figure 8H–L). These data suggested that Nrf2 was the key target for SS-31 to play inhibitory roles in NLRP3 inflammasome activation and pulmonary fibrosis.

## 4. Discussion

IPF is a severe progressive lung disease with poor survival and limited curative therapies [29]. Currently, only two drugs, i.e., the potent tyrosine kinase inhibitor nintedanib and the pyridone derivative pirfenidone, are FDA-approved treatments for IPF, but unfortunately, they have been found to improve a few outcomes and cause frequent side effects [30]. Hence, developing safe and applicable therapeutic candidates for IPF is urgently needed.

In the present study, we first demonstrated that SS-31 can alleviate BLM-induced pulmonary fibrosis in mice. Abundant evidence, including our previous publication, has shown that the inflammatory response controlled by macrophages is the crucial pathogenesis of pulmonary fibrosis [12,31]. Here, we found that SS-31 treatment inhibited lung inflammation with reduced expression of IL-1β and IL-18 in vivo. Further study revealed that SS-31 suppressed the NLRP3 inflammasome by restoring Nrf2 signaling and mitochondrial function in macrophages to contribute to the decreased production of the cytokines IL-1β and IL-18. These data supported the effective function and provided the molecular mechanism of SS-31 in protecting against IPF.

SS-31 is a novel mitochondria-targeting antioxidant that is small, easy to synthesize, and water-soluble [32]. It has received extensive attention because it protects and restores the structure and function of damaged mitochondria with no effects on healthy mitochondria [33]. A number of studies have demonstrated that it can be taken up not only by cells but also transported across cells, thus being rapidly absorbed after administration. Considering its low cytotoxicity and mitochondrial targeting, SS-31 has been found to protect against various diseases, including cardiac, neurological, kidney, and aging-related diseases [34,35,36]. Moreover, the safety and tolerability of therapeutic SS-31 were confirmed in some clinical trials, providing a basis for its widespread application [37]. However, to date, the role of SS-31 in IPF has never been reported, and our current study suggests that SS-31 might be an attractive candidate for the treatment of IPF.

Macrophages are the most abundant immune cells and are central to homeostasis in the lungs. Over decades of study, macrophages have been implicated in the key pathogenesis of pulmonary fibrosis. On the one hand, they produce and secrete various cytokines, including both M1 markers (TNF-α, IL-1β, IL-6, and so on) and M2 markers (IL-4, IL-13, TGF-β, and so on), deteriorating epithelial injury and fueling the inflammatory processes linked to fibrosis [11,38]. On the other hand, interactions between macrophages and other cells, such as epithelial cells and fibroblasts, can contribute to fibrogenesis by promoting epithelial–mesenchymal transition and myofibroblast differentiation [39,40]. Therefore, strategies that suppress macrophage activation might be potentially effective strategies for IPF treatment. However, in IPF, it has been suggested that both M1-like and M2-like macrophages coexist according to the complicated microenvironment and contribute to pulmonary fibrosis via multiple pathways [10,41]. Here, by simulating the complicated immune condition during IPF development in which macrophages are located, we induced macrophages using LPS together with IL-4 or IL-33, another key cytokine we demonstrated in our previous work, to further explore the cellular mechanism of SS-31. Our results confirmed that SS-31 significantly reduced the production of IL-1β and IL-18 by macrophages in response to the above stimuli, coupled with alleviating inflammation in the early stage (3 and 7 days) after bleomycin challenge. These data supported that the anti-fibrotic function of SS-31 might be attributed to its suppressed function on macrophage activation and subsequently reduced production of IL-1β and IL-18.

The NLRP3 inflammasome is a key cytosolic protein complex responsible for the release of IL-1β and IL-18 [42]. It consists of the innate immune receptor protein NLRP3, adapter protein ASC, and inflammatory protease caspase-1, facilitating innate immune defense and homeostatic maintenance [43]. Previous studies in animal models and patients with pulmonary fibrosis have found that IL-1β and IL-18 levels were significantly elevated in BALF and BALF macrophages, consistent with pre-existing NLRP3 inflammasome activation [44]. Recently, studies revealed that mitochondria are crucial regulators of the NLRP3 inflammasome [45]. Impaired mitochondria could emit signals including excess mtROS, cytosolic translocation of mtDNA, or relocation of mitochondria, all of which have been identified to serve as direct activators of the NLRP3 inflammasome [46]. Thus, we hypothesized that SS-31 reduced the expression of IL-1β and IL-18 by suppressing NLRP3 inflammasome activation by regulating mitochondrial function. As expected, our data showed that SS-31 treatment significantly reduced the mRNA and protein levels of NLRP3, ASC, and caspase-1 in mice with BLM administration and macrophages under IL-33 or LPS/IL-4 induction, accompanied by decreased production of ROS and mtDNA. The results suggested that SS-31 exerted its antifibrotic function by restoring mitochondrial dysfunction and inhibiting NLRP3 inflammasome activation.

During oxidative stress, Nrf1 and Nrf2 emerge as the critical downstream mechanisms involved in mitochondrial dysfunction [47]. They are not only linked to the expression of many genes required for mitochondrial respiratory function but also play critical roles in enhancing mtDNA levels, oxidative phosphorylation (OXPHOS) activity, and mitochondrial protein import and assembly [48]. However, genetic studies show that Nrf1−/− is embryonic lethal, while Nrf2 is dispensable for the development of mice, clearly demonstrating that these two genes have unique target genes despite sharing overlapping ones [49,50]. Accumulating evidence shows that both Nrf1 and Nrf2 play important roles in respiratory function. For example, NRF1 knockdown can alleviate LPS-triggered lung inflammatory injury by regulating the GSK-3β/β-catenin pathway [51], NRF-1 can protect against pulmonary fibrosis by binding to the promoters of TGFβ1 repressors [52], and Nrf2 alleviates lung injury via various mechanistic pathways, such as inhibiting excessive autophagy and ROS production [53]. In our present study, both Nrf1 and Nrf2 were examined, and the results showed that only the expression of Nrf2 was dramatically upregulated with SS-31 treatment in the lungs of BLM-induced mice and macrophages with IL-33 or LPS/IL-4 stimulation. To further confirm whether the role of SS-31 in protecting against fibrosis was dependent on Nrf2 signaling alteration in macrophages, we explored the effects of SS-31 on Nrf2−/− macrophages under stimuli and mice with BLM challenge. The results showed no significant difference in fibrosis or NLRP3 inflammasome activation between control and SS-31 -treated Nrf2-deficient mice and macrophages, which was consistent with the hypothesis that Nrf2 plays an essential role in SS-31-mediated attenuation of fibrosis.

## 5. Conclusions

SS-31 treatment significantly attenuated pulmonary fibrosis and inflammation induced by BLM, with reduced expression of IL-1β and IL-18. In terms of the mechanism, SS-31 protected against IPF by inhibiting NRF2-mediated NLRP3 inflammasome activation in macrophages. These results provide a potential agent for the treatment of pulmonary fibrosis. Our article also has some limitations: 1. During the experiment, the overall state, hair, respiration, body weight, and blood biochemical results of the mice were not reduced, and dynamic monitoring and statistics increased the uncertainty in the experimental results. 2. In this article, we found that the treatment of SS-31 affects the expression of inflammatory factors through Nrf2 but did not further elucidate its mechanism. Further research may explore deeper mechanisms using keap1 activators or Nrf2 inhibitors. 3. We only conducted studies at the animal level in this article. In the future, we will collect some clinical samples to further explore our results.

## Data Availability

The datasets used during the current study are available from the corresponding author upon reasonable request.
